# Effects of a Rotating Cone on the Mixed Convection in a Double Lid-Driven 3D Porous Trapezoidal Nanofluid Filled Cavity under the Impact of Magnetic Field

**DOI:** 10.3390/nano10030449

**Published:** 2020-03-02

**Authors:** Ali J. Chamkha, Fatih Selimefendigil, Hakan F. Oztop

**Affiliations:** 1 Department of Mechanical Engineering, Prince Sultan Endowment for Energy and Environment, Prince Mohammad Bin Fahd University, Al-Khobar 31952, Saudi Arabia; achamkha@pmu.edu.sa; 2 Department of Mechanical Engineering, Celal Bayar University, 45140 Manisa, Turkey; 3 Department of Mechanical Engineering, Technology Faculty, Firat University, 23119 Elazig, Turkey; hfoztop1@gmail.com

**Keywords:** rotating cone, MHD flow, finite element method, nanofluid, 3D cavity, porous media

## Abstract

Effects of a rotating cone in 3D mixed convection of CNT-water nanofluid in a double lid-driven porous trapezoidal cavity is numerically studied considering magnetic field effects. The numerical simulations are performed by using the finite element method. Impacts of Richardson number (between 0.05 and 50), angular rotational velocity of the cone (between −300 and 300), Hartmann number (between 0 and 50), Darcy number (between 10${}^{-4}$ and $5\times 10^{-2}$), aspect ratio of the cone (between 0.25 and 2.5), horizontal location of the cone (between 0.35 H and 0.65 H) and solid particle volume fraction (between 0 and 0.004) on the convective heat transfer performance was studied. It was observed that the average Nusselt number rises with higher Richardson numbers for stationary cone while the effect is reverse for when the cone is rotating in clockwise direction at the highest supped. Higher discrepancies between the average Nusselt number is obtained for 2D cylinder and 3D cylinder configuration which is 28.5% at the highest rotational speed. Even though there are very slight variations between the average Nu values for 3D cylinder and 3D cone case, there are significant variations in the local variation of the average Nusselt number. Higher enhancements in the average Nusselt number are achieved with CNT particles even though the magnetic field reduced the convection and the value is 84.3% at the highest strength of magnetic field. Increasing the permeability resulted in higher local and average heat transfer rates for the 3D porous cavity. In this study, the aspect ratio of the cone was found to be an excellent tool for heat transfer enhancement while 95% enhancements in the average Nusselt number were obtained. The horizontal location of the cone was found to have slight effects on the Nusselt number variations.

## 1. Introduction

Mixed convection in cavities due to moving surfaces has been a subject of many important heat transfer applications in electronic cooling, convective drying, solar power, some chemical engineering processes, and many others [1,2]. The interaction of the forced flow and other effects such as natural convection will be made even more complex by including magnetic field effects and complicated geometries. There are many investigations that simplified the thermal engineering problem in two dimensional cavities with simple shapes. However, in real engineering problems considering three dimensional geometry is more realistic and two dimensional modeling may not be adequate to represent the three dimensional fluid flow features. In the current work, mixed convection in a three dimensional trapezoidal cavity double lid driven which accounts for geometrical non-uniformity is also considered.

In convective heat transfer applications in cavities, many active and passive heat transfer enhancement techniques are offered. In one of these methods, stationary or rotating circular cylinders are recently used in many applications [3,4,5,6,7,8]. The rotating cylinders immersed in fluid in cavities have many applications, such as in rotating tube heat exchangers, drilling of oil wells, rotating shafts, and many others. Rotational speed, size, location, and thermal conductivity are among the most important parameters that can be considered for heat transfer enhancement. These effects were found to be effective in thermal performance for an inner rotating cylinder in a differentially heated cavity problem as studied in ref. [9]. In the numerical work of Hussain and Hussein [10], mixed convection in a two dimensional cavity with an inner rotating cylinder is studied by using the finite volume method and it was observed that the location of the cylinder has significant impacts on the convection enhancement. Ghaddar and Thiele [11] used spectral element method for analyzing the effects of a rotating cylinder in an isothermal cavity for considering various rotational speeds of the cylinder while heat transfer was found to enhance with rotation of the cylinder at low Rayleigh number. Fu et al. [12] examined the convection in a cavity with rotating cylinder effects by using penalty finite-element method. It was found that the direction of the rotation contributes significantly to the heat transfer enhancement. In the literature, there are a few studies that considered the impact of rotating cylinders within cavities on convection for three dimensional configurations [13]. In the work of Kareem and Gao [14], three dimensional mixed convection in a differentially heated cavity containing an inner adiabatic rotating cylinder was simulated for the range of non-dimensional rotational speeds of −5 and 5 in the turbulent flow regime. Heat transfer increment was observed with rotation but their impacts on the different surfaces was found to be different. Selimefendigil and Oztop [15] examined the impacts of two rotating inner circular cylinders in three dimensional cavity for steady, laminar flow regime. Both enhancement and deterioration of average heat transfer rate are observed depending upon the rotational direction of the cylinders.

Magnetic field effects are recently used for convective heat transfer control [16,17,18,19]. The effects of magnetic field are encountered in geothermal energy extraction, glass float, coolers of nuclear reactors and many others. For convection in cavities, the magnetic field was found to reduce the heat transfer rate [20,21,22]. However, recent studies showed that the magnetic field effects can be beneficial to enhance the convection for configurations that produce multiple re-circulations as in vented cavities [23] or in separated flows as encountered in sudden area expansion geometries [24,25]. Magnetic field effects are recently used with nanofluids [26,27,28,29]. The technology of nanofluids were successfully implemented in various technological applications related to thermal science such as in solar power, thermal energy storage, refrigeration, thermal management and convective heat transfer control [30,31,32,33,34,35,36,37]. Convective heat transfer in 3D cavities with nanofluid considering magnetic field effects were performed by several researchers [38,39]. Sheikholeslami et al. [40] numerically studied the effects of Lorentz forces in 3D cavity with nanofluids and the numerical results showed that the heat transfer rate is reduced with higher magnetic field strength. In another study, Al-Rashed et al. [41] used finite volume method for analyzing 3D natural convection in a cubic enclosure with carbon nano-tube (CNT)-water nanofluid and magnetic field. The average Nu number was found to reduce by 50% when magentic field strength is increased from Hartmann number of 50 to Hartmann number of 100. In a recent work, Ghasemi and Siavashi [42] examined the magneto-hydrodynamic Cu-water nanofluid in a three-dimensional cavity with moving surfaces bu using the MRT- lattice Boltzmann method. The negative impact of Hartmann number on heat transfer rate was observed but it was noted that magnetic field can be aligned such that negative impact can be reduced. Magnetic field effects with nanofluid are also used in many porous media applications [43,44,45,46,47]. Convection in porous media may find important application areas such as in solar collectors, solidification, thermal insulation and many others.

In the present study, a rotating cone is used in three dimensional mixed convection of double lid driven trapezoidal porous enclosure considering magnetic field effects with CNT-water nanofluid. These particles were found to be very promising in the heat transfer enhancement in comparison with the other nanoparticles [48,49]. A rotating cone which can be considered to be a generalization of a rotating cylinder is used in 3D cavity which adds novelty to the current configuration. The aspect ratio of the eccentric cone can be adjusted along with other parameters encountered in rotating cylinder such as rotational speed, size and location. There are a few studies of mixed convection in 3D cavities with rotating cylinder in cavities; however, it is the first time a rotating cone is used in a 3D double lid-driven porous cavity. Owing to the diversity in the application of mixed convection in lid-driven cavities for many thermal engineering problems, use of magnetic field with very highly conductive nanoparticles and using a rotating cone provide promising multiple methods for convective heat transfer control in many heat transfer engineering problems.

## 2. Modeling Steps in the Numerical Simulation

### 2.1. Geometric Model and Governing Equations

**Figure 1:**
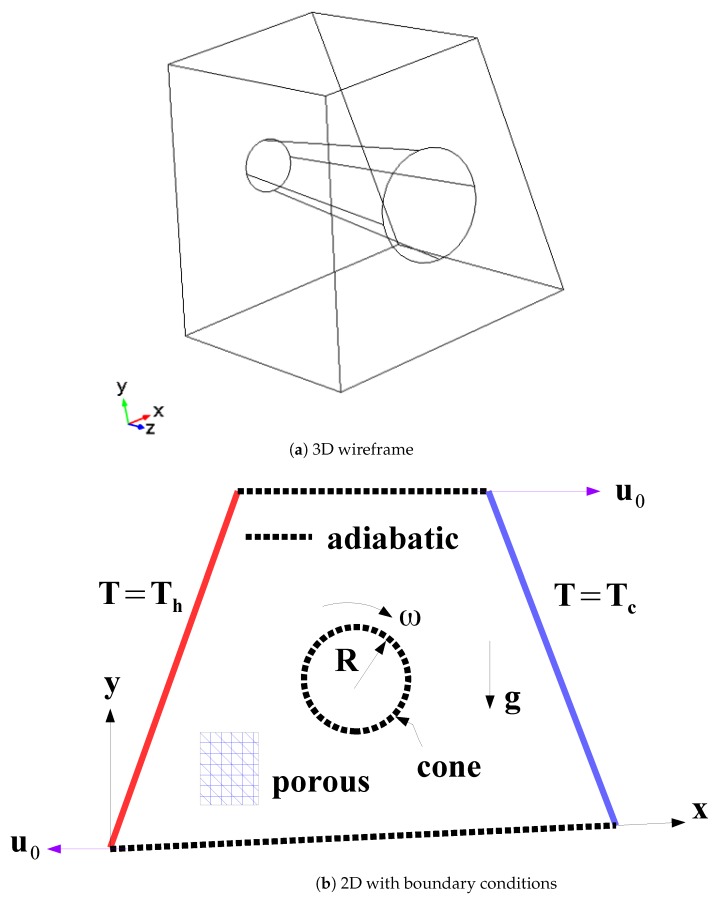
Schematic description of the physical model (**a**) 3D (**b**) 2D with boundary conditions.

A schematic view of the 3D representation and 2D view with boundary conditions are shown in Figure 1. A trapezoidal 3D cavity with side surface inclination of 10${}^{\circ }$ and size H is considered. A rotating cone is located in the mid of the cavity with rotational speed of ω. r1 and r2 are the radius of the base surfaces which are circular and AR denotes the aspect ratio as AR = r1/r2. The upper and lower horizontal surface of the cavity are moving with constant speeds of u0 in the positive and negative × directions. The cavity side surfaces are at fixed temperatures of T${}_{h}$ and T${}_{c}$ with T${}_{h}$> T${}_{c}$. Other surfaces of the 3D cavity and surfaces of the rotating cone are adiabatic. As the heat transfer fluid SWCNT-water nanofluid is used considering the impacts of magnetic field and the properties are shown in Table 1. The base fluid Prandtl number is 6.9. The fluid is incompresible and Newtonian. Laminar, steady and three dimensional flow assumptions are used. The viscous dissipation and radiation effects are also not taken into account. The Brinkman-extended Darcy porous model is used.

The conservation equations are written in compact notation as:

(1) $$\nabla .\vec{U}=0$$

(2) $$\frac{1}{\varepsilon^{2}}\left(\vec{U}.\nabla \right)\vec{U}=-\frac{1}{\rho_{nf}}\nabla P+\frac{\nu_{nf}}{\varepsilon }\nabla^{2}\vec{U}+\vec{g}\beta_{nf}\left(T-T_{c}\right)-\frac{\nu }{\kappa }\vec{U}+\frac{\sigma_{nf}}{\rho_{nf}}\left(\vec{U}\times \vec{B}\right)\times \vec{B}$$

(3) $$\left(\vec{U}.\nabla \right)T=\alpha_{nf}\nabla^{2}T$$

The inclusion of CNT particles affects the variation of electrical conductivity along with the other thermophysical properties. The magnetic Reynolds number is much smaller than one and the induced magnetic field effects are not taken into account. The magnetic field is assumed to be uniform throughout the computational domain. A transverse magnetic field which is uniform and parallel to the z-axis is used. Joule heating effects are neglected along with the electric field and induced magnetic effects. The last term in the above given momentum equation are the Lorentz forces. Based on those simplifications, $-\frac{\sigma_{nf}}{\rho_{nf}}B^{2}u$ and $-\frac{\sigma_{nf}}{\rho_{nf}}B^{2}v$ become the x and y contributions of the Lorentz forces.

The dimensional boundary conditions are given as:

- For horizontal upper moving surface, $u=u_{0},\;v=w=0\;\frac{\partial T}{\partial n}=0$.
- For left side surface, $u=v=w=0\;T=T_{h}$.
- For right side surface, $u=v=w=0\;T=T_{c}$.
- For horizontal lower moving surface, $u=-u_{0},\;v=w=0\;\frac{\partial T}{\partial n}=0$.
- Along the surface of the cone, $u=-\omega \left(y-y_{c}\right),\;v=\omega \left(x-x_{c}\right),\;w=0\;\frac{\partial T}{\partial n}=0$.

(4) Pr=νfαf,Gr=gβf(Th−Tc)H3νf2,Ha=HB0σfμf,Da=κH2,Re=u0Hν2,Ri=GrRe2

Thermal performance of the system is evaluated by using the Nusselt numbers. The local and average Nusselt number for the hot surface are calculated as in the following:

(5) $${\mathrm{Nu}}_{s_{1},s_{2}}=-\frac{k_{nf}}{k_{f}}\left(\frac{\partial \theta }{\partial S}\right),\;\;{\mathrm{Nu}}_{m}=\frac{1}{S_{1}S_{2}}\int_{0}^{S_{1}}\int_{0}^{S_{2}}{\mathrm{Nu}}_{s_{1},s_{2}}ds_{1}ds_{2}.$$

The solution of the equations is made by using the Galerkin weighted residual finite element method. In the formulation, the flow variables are approximated by using the interpolation functions:

(6) $$\begin{array}{cc} \; & u=\sum_{k=1}^{N^{u}}\Psi_{k}^{u,v,w}U_{k},\;v=\sum_{k=1}^{N^{v}}\Psi_{k}^{u,v,w}V_{k},\;w=\sum_{k=1}^{N^{w}}\Psi_{k}^{u,v,w}W_{k}\\ \; & p=\sum_{k=1}^{N^{p}}\Psi_{k}^{p}P_{k},\;\;T=\sum_{k=1}^{N^{u}}\Psi_{k}^{T}T_{k}. \end{array}$$

$\Psi^{u,v,w},\Psi^{p}$ and $\Psi^{T}$ are the shape functions for field variables. U,V,P and *T* denote the values of the respective variables at the nodes of the element. The residuals are set to be zero as:

(7) $$\int_{\Omega }F_{k}\left(x\right)Rdv=0.$$

$F_{k}$ is the weight function. The Newton-Raphson method was used for the solution of nonlinear residual equations.

### 2.2. CNT-Water Nanofluid Property Equations

CNT-water nanofluid effective thermo-physical relations are given as [50]:

(8) $$\rho_{nf}=\left(1-\phi \right)\rho_{f}+\phi \rho_{p}$$

(9) $${\left(\rho c_{p}\right)}_{nf}=\left(1-\phi \right){\left(\rho c_{p}\right)}_{f}+\phi {\left(\rho c_{p}\right)}_{p}$$

(10) $${\left(\rho \beta \right)}_{nf}=\left(1-\phi \right){\left(\rho \beta \right)}_{f}+\phi {\left(\rho \beta \right)}_{p}.$$

A correlation which considers the space distribution of the CNTs in the nanofluid is used for thermal conductivity. It is defined as [51]:

(11) $$\frac{k_{nf}}{k_{f}}=\frac{\left(1-\phi \right)+2\phi \frac{k_{CNT}}{k_{CNT}-k_{f}}\ln\frac{k_{CNT}+k_{f}}{2k_{f}}}{\left(1-\phi \right)+2\phi \frac{k_{f}}{k_{CNT}-k_{f}}\ln\frac{k_{CNT}+k_{f}}{2k_{f}}}$$

This definition of the thermal conductivity is shown to produce accurate results when experimental results are used [51].

The Brinkman model is chosen for the dynamic viscosity of the nanofluid [52]:

(12) $$\mu_{nf}=\frac{\mu_{f}}{{\left(1-\phi \right)}^{2.5}}$$

This model does not take into account the temperature dependence and size of the particle effects. It has been used in various studies for convective heat transfer applications and the heat transfer fluid was considered to be Newtonian up to a specified values of solid particle volume fraction [53,54]. In the experimental work of Halelfadl et al. [55], the viscosity of CNT-water was examined and temperature and solid particle volume fraction effects were analyzed. It was observed that the fluid behaves non-Newtonian (shear thinning) for higher nanoparticle volume fractions. In the analytical work of Benos et al. [56], impacts of CNT aggregations were included in the viscosity and thermal conductivity of CNT-water nanofluid. In a recent work, molecular dynamics simulation method was used to obtain the viscosity of a model water-based nanofluid with single walled CNT [57]. A correlation for the volume fraction in the range of 0.25% and 0.65% is offered and comparisons are made between various available models for the viscosity of nanofluids.

For the definition of electrical conductivity of CNT-water nanofluid, Maxwell’s model was used [51]:

(13) $$\frac{\sigma_{nf}}{\sigma_{f}}=1+\frac{3\phi \left(\frac{\sigma_{p}}{\sigma_{f}}-1\right)}{\left(\frac{\sigma_{p}}{\sigma_{f}}+2\right)-\phi \left(\frac{\sigma_{p}}{\sigma_{f}}-1\right)}$$

### 2.3. Mesh Examination and Code Verification

Mesh is composed of tetrahedral elements and its independence is assured by using various number of elements. Table 2 shows the average Nusselt number variations versus number of elements considering three values of Richardson numbers. G6 with 69,769 number of elements is used for the subsequent computations. Validation of the present work is made by using different available numerical studies in the literature. In the first study, mixed convection in a double lid-driven cubic enclosure is examined for various Richardson and Reynolds numbers as studied in ref. [58]. The average Nusselt numbers of the configurations with (Re = 100, Ri = 1) and (Re = 100, Ri = 10) are 1.70 and 1.20 in ref. [58] whereas these values are calculated as 1.63 and 1.18 with the present solver. Another validation is conducted by using the experimental results of Heyhat et al. [59]. In this work, forced convection of Al${}_{2}$O${}_{3}$-water nanofluid in a horizontal tube in laminar flow conditions was examined for nanofluid solid volume fraction up to 2%. Following property relations for the thermal conductivity and dynamic viscosity are considered:

(14) $$k_{nf}=k_{bf}\left(T\right)\left(1+8.733\phi \right),\;\;\;\mu_{nf}=\mu_{bf}\left(T\right)\exp\left(\frac{5.989\phi }{0.278-\phi }\right).$$

**Figure 2:**
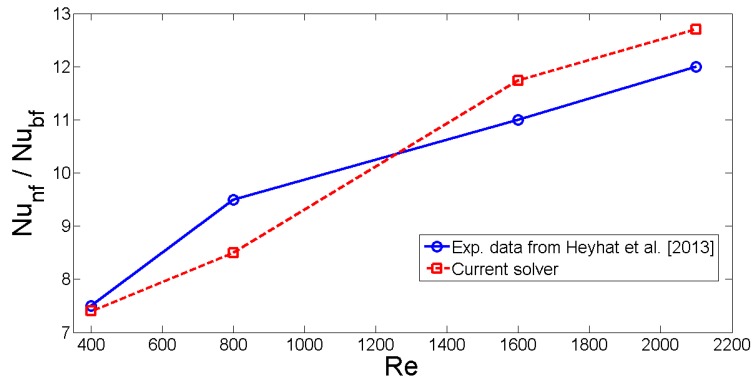
Ratio of the Nusselt number of nanofluid to pure water obtained in the experimental work in Ref. [59] and calculated with the current solver.

Temperature dependence (between 20${}^{\circ }$ and 60${}^{\circ }$) of the properties are considered. The ratio of Nusselt numbers for nanofluid and water case is shown in Figure 2 for different values of Reynolds numbers. Highest deviation is obtained as 9.40% between the experimental data and present solver at Reynolds number of 800.

**Figure 3:**
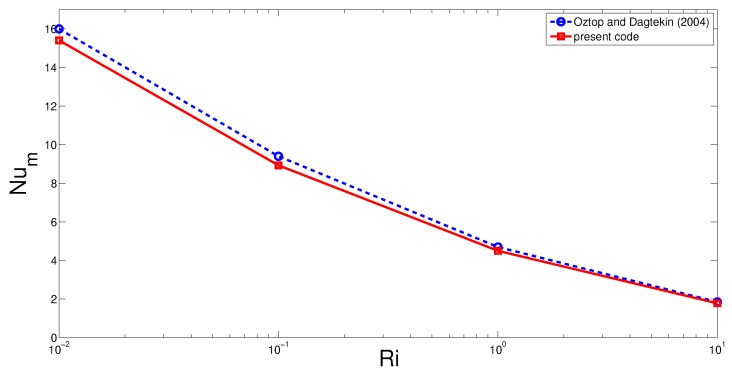
Average Nusselt number comparisons for various Richardson numbers for a double lid driven cavity problem (opposite lid velocity direction) computed in ref. [60] and obtained with the current solver.

Final verification of the code is made by using the results from the two dimensional double-lid driven cavity problem analyzed in ref. [60]. Figure 3 shows the average Nusselt number comparisons for various Richardson numbers.

The results of the validation studies show that the current solver can predict the nanofluid behavior and convective heat transfer of lid driven cavity problems in 2D and 3D configurations.

## 3. Results and Discussion

In the present study, impacts of a rotating cone on mixed convection of CNT-water nanofluid in a 3D trapezoidal porous cavity are examined considering magnetic field effects.

**Figure 4:**
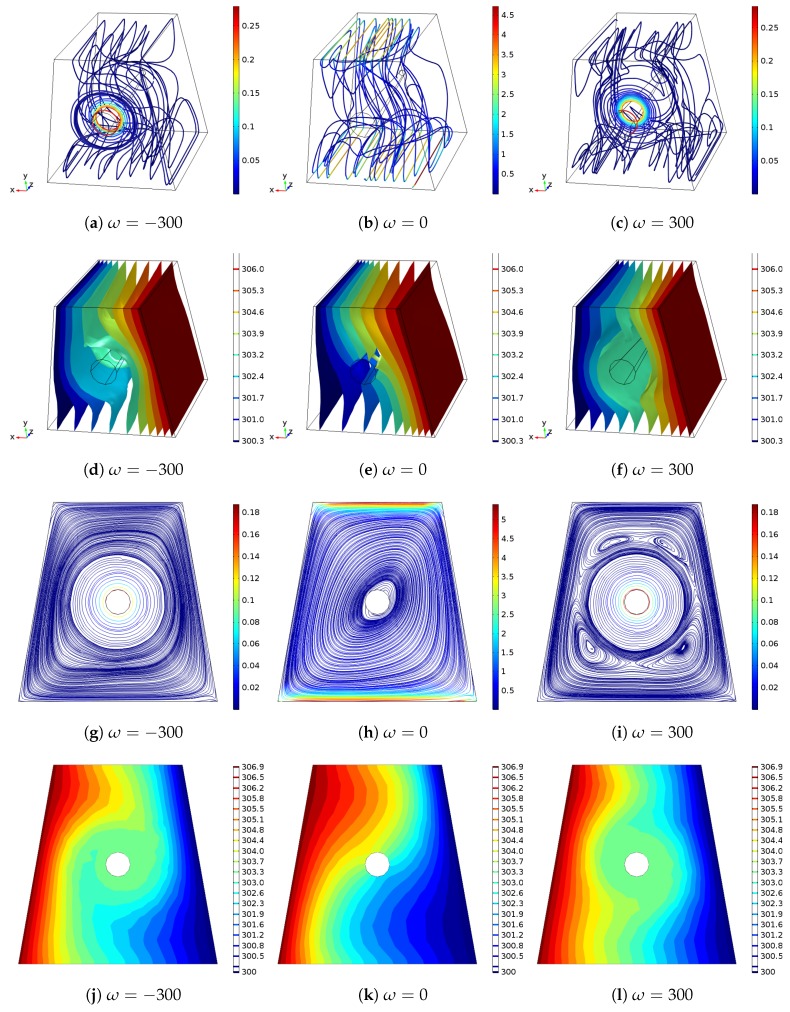
Effects of angular rotational speed of the rotating cone on the variation of flow and thermal patterns for 3D and 2D mid-plane of the cavity (Ri = 5, Ha = 10, Da = 10${}^{-3}$, AR = 0.25, x${}_{0}$ = 0.5 H, ϕ=0.04).

Richardson number is the ratio of the free convection effects to the forced convection due to the moving surface. The Rayleigh number is fixed to the 10${}^{5}$ and a lower Richardson number value gives a higher velocity of the upper and lower moving surfaces. As the value of Ri rises, the natural convection effects are increased while the penetrating fluid motion form the moving surfaces are reduced. Impact of rotational speed of the cone on the 3D and 2D mid-plane flow and thermal patterns are shown in Figure 4 (Ri = 5, Ha = 10, Da = 10${}^{-3}$, AR = 0.25, x${}_{0}$ = 0.5 H, ϕ=0.04). As compared to stationary cylinder case, multi-recirculation regions are established for ω=−300 while the diagonally elongated vortex becomes flattened for ω=300. The thermal gradients become higher especially for the upper part of the hot surface with rotation of the cone.

**Figure 5:**
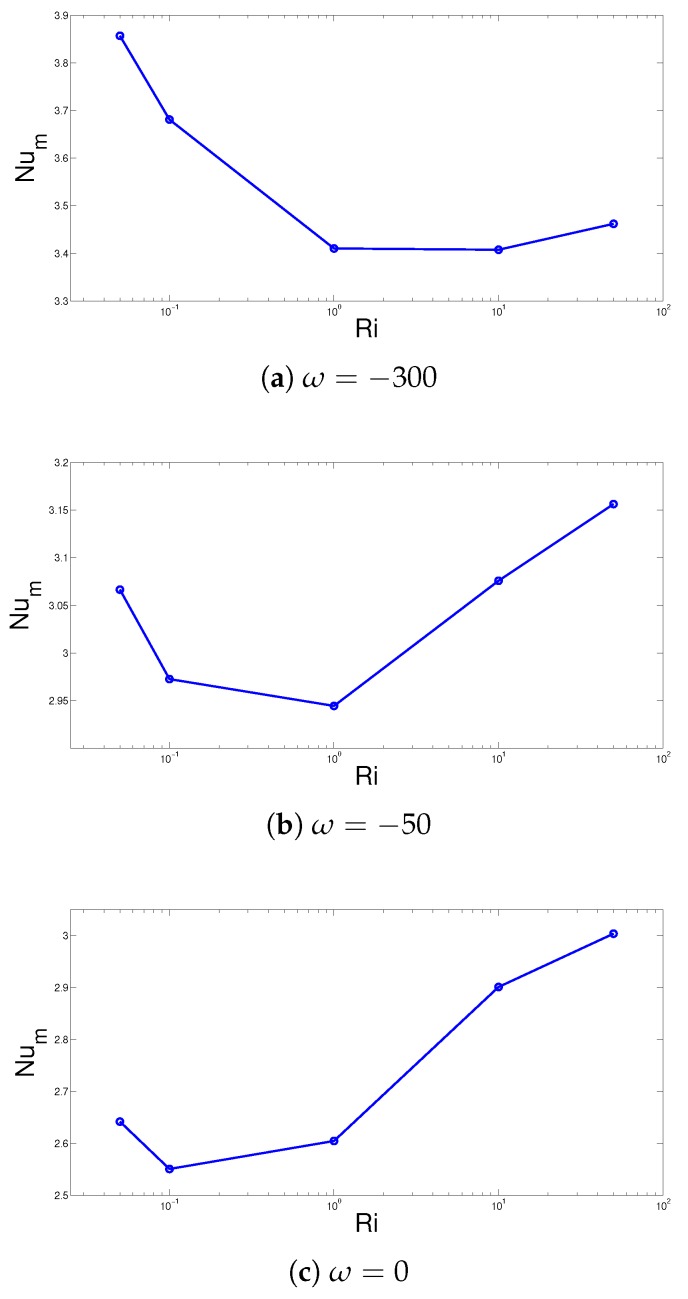

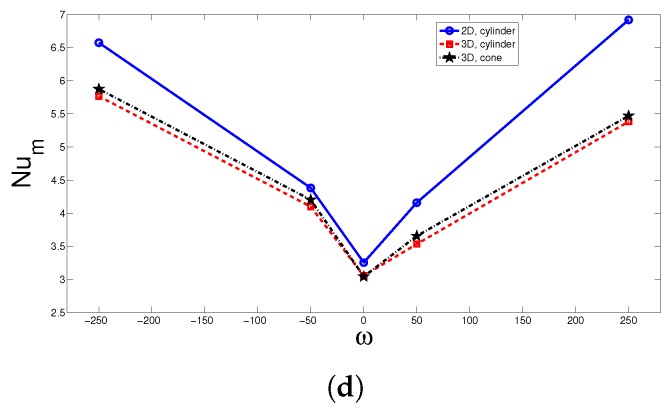
The average Nusselt number versus Richardson number variations for different angular rotational speeds (**a**–**c**) (Ha = 10, Da = 10${}^{-3}$, AR = 0.25, x${}_{0}$ = 0.5 H, ϕ=0.04) and variation of average Nusselt number with respect to changes in ω for 2D circular cylinder, 3D circular cylinder and 3D circular cone (AR = 2) configurations (**d**) (Ha = 10, Da = 10${}^{-3}$, ω=−100, x${}_{0}$ = 0.5 H, ϕ=0.04).

The average Nusselt number variations with respect to changes in the Ri number for three values of angular rotational speed of the cylinder are shown in Figure 5a–c. The average heat transfer behavior for various Richardson numbers are significantly affected by the angular rotational velocity of the cone. For stationary cone case at ω=0, the average Nusselt number generally enhances with higher values of Ri numbers. However, at the highest speed of ω=−300, the impact is reverse. This could be attributed to reduced convective effects of the rotating cone with higher velocities of the upper and lower surfaces which resulted in heat transfer deterioration. The variation of the average Nu value of the hot surfaces with varying values of ω of the objects (2D cylinder, 3D cylinder and 3D cone) are shown in Figure 5d. The average Nu value is higher for 2D cylinder case and discrepancies between 2D and 3D configuration and the average Nusselt number values rise with higher ω. There is only 7% higher values are achieved for 2D cylinder case as compared to 3D cylinder configuration while this value becomes 14% and 28.5% for rotational speeds of ω=−250 and ω=250, respectively. There are some negligible variations in the average Nusselt number for 3D cylinder and 3D cone and the highest variation is 2% at ω=−250.

In the current work, the competing forces between the magnetic field and hydrodynamic forces can be defined by using additional interaction parameters. An additional parameter which is the interaction index (N${}_{L}$) can be defined as:

(15) NL=Ha2Re.

It gives the ratio of the Lorentz forces to the inertia forces due to the moving wall. In the present work, the rotation of the cone also affects the convective flow features. Another interaction parameter which defines the ratio of the Lorentz forces to the rotational effects of the cone can be defined as:

(16) Nω=Ha2Reω.

and the rotational Reynolds number is defined as:

(17) Reω=ωD2νf.

The value of N${}_{L}$ corresponds to 0, 0.185 and 4.64 at Richardson number of 0.05 while they are 0, 5.87 and 146.84 at Richardson number of 50 when Hartmann number values of Ha = 0, Ha = 10 and Ha = 50 are considered. When rotation of the cone is considered at fixed value of Ri=1, the interaction parameter $N_{\omega }$ takes values of 0, 10.59 and 251.48 for rotational speed of 10 while these values become 0, 0.4 and 10.06 for rotational speed of 250 when Hartmann number of Ha = 0, Ha = 10 and Ha = 50 are considered.

**Figure 6:**
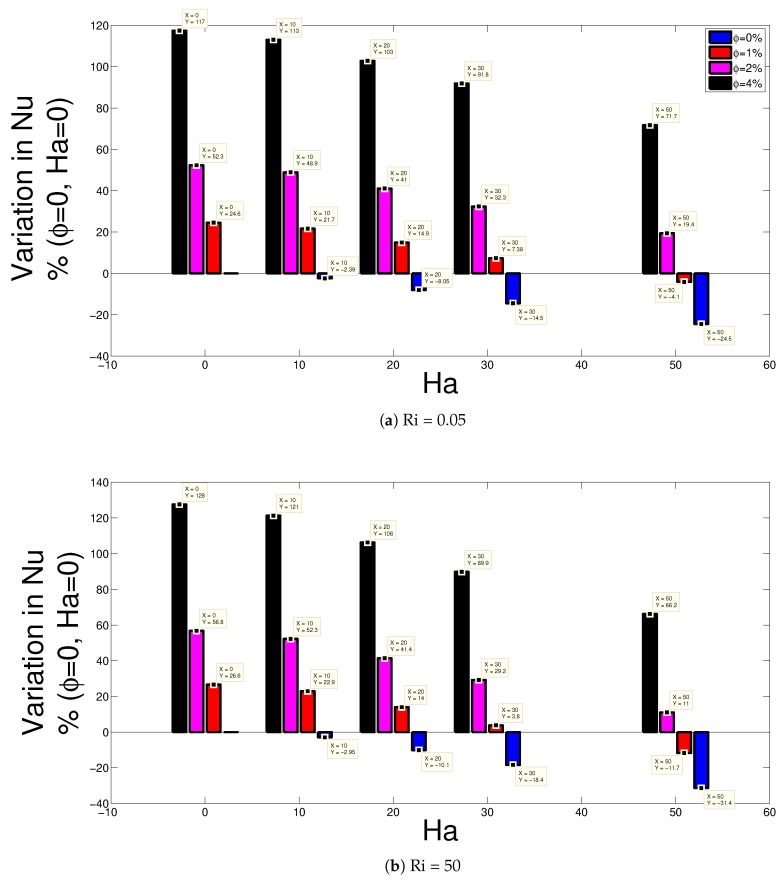
Enhancement in the average Nusselt number with respect to changes in ϕ and Hartmann number considering Richardson number of 0.05 (**a**) and Richardson number of 50 (**b**) (Da = 10${}^{-3}$, AR = 0.25, x${}_{0}$ = 0.5 H).

In the current work, nanofluids are used with magnetic field effects. In the experimental work of Kaneda et al. [61], where natural convection for a liquid metal with uniform magnetic field is examined, the convective heat transfer was found to be reduced with larger magnetic field strength. In another experimental work, free convection of a magnetic fluid in the annular space of two horizontal cylinders was examined [62]. It was observed that the direction and amplitude of magnetic field were effective in convective heat transfer and magnetic field can be used as a control tool. Convective heat transfer features around a heated wire under uniform magnetic field and magnetic field gradient were experimentally conducted in Ref. [63]. The orientation and strength of the magnetic field were shown to play a significant role on the heat transfer features. Both the thermal and electrical conductivity of the base fluid changes by introducing nano sized particles. The configuration with water (ϕ=0) and without magnetic field effects (Ha = 0) are taken as the reference case while enhancement or deterioration the average Nu value for various nanofluids with different solid particle volume fractions considering two values of Richards numbers are shown in Figure 6. The amount of heat transfer enhancement with nanoparticle addition reduces for higher Hartmann number case for all Ri numbers. Similar results have also been in previous studies for convective heat transfer studies in cavities considering magnetic field effects. Deterioration of the heat transfer is achieved after Ha = 10 for ϕ=0 and at Ha = 50 for ϕ=1% considering all Richards number cases. The average enhancement of heat transfer rate in the absence of magnetic field is highest for Ri = 1 and the amount is 133% while at Ha = 50, this value is reduced to 84.3%. It is observed that the heat transfer enhancement is still very high with the inclusion of CNT nanoparticles even in the presence of destructive effects of MHD on convection. In the experimental work of Sarafraz et al. [64], performance of COOH functionalized multi-walled carbon nanotubes-water nanofluid was experimentally tested for a double pipe heat exchanger. Small penalty was noted for pressure drop while significant enhancements in the thermal performance up to 44% for the highest mass concentration of wt.%=0.3 were observed. In another experimental work, thermal performance features of CNT-water nanofluid in a tube with inserted helical screw louvered rods were analyzed for solid volume fractions of 0.1%, 0.2%, and 0.5% in Ref. [65]. The highest thermal performance index of 1.23 was obtained for 0.5% volume concentration for a twist ratio of 1.78. From these experimental studies and form numerical studies as mentioned above, it is obvious that using CNT nanoparticles in heat transfer fluids gives higher thermal performance.In the current work, the highest amount of average heat transfer reduction with magnetic field in the absence of nanoparticles is 31.4% at Richardson number of 50.

**Figure 7:**
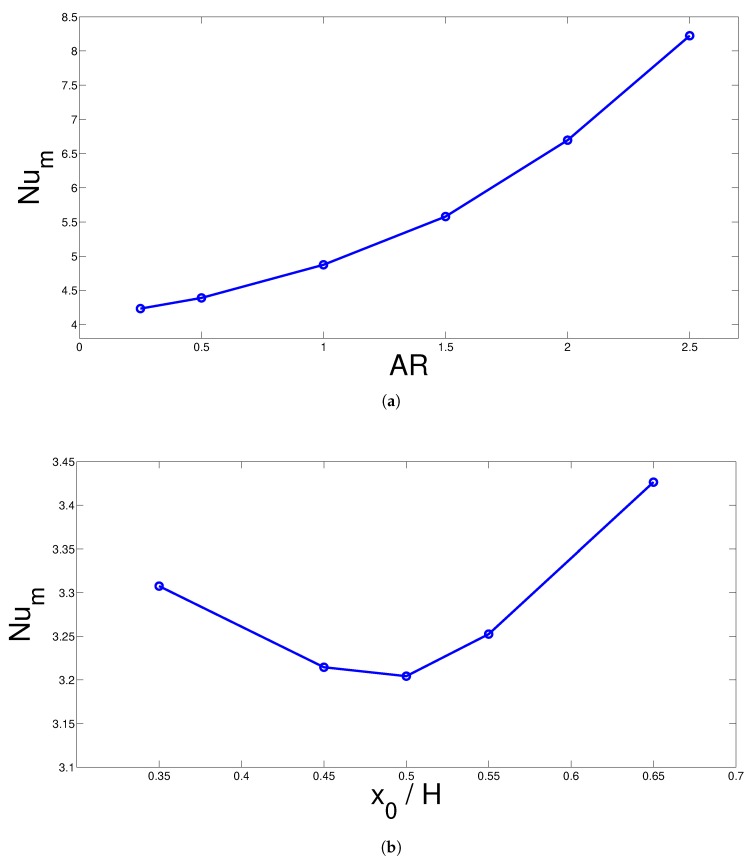
Effects of aspect ratio (**a**) and x-location of the rotating cone (**b**) on the variation of average Nusselt number of the hot surface (Ri = 5, Ha = 10, Da = 10${}^{-3}$, ϕ=0.04).

The aspect ratio and location of the rotating cone can be considered other parameters that could contribute to the convective heat transfer enhancement. Aspect ratio of the cone denotes the ratio of the radius of the base circular surfaces of the cone. A higher aspect ratio denotes a higher average radius of the cone. As the value of AR is higher, the gap between the surfaces of the hot wall and rotating cone reduces. Higher impact of the convective flow motion due to the rotating cone is obtained with higher AR and thermal gradients near the hot surface are expected to become steepened. Impacts of AR and vertical location of the rotating cone on the average variation of the Nusselt number of the hot surface are shown in Figure 7. AR = 1 denotes a rotating circular cylinder in 3D cavity configuration. The average Nusselt number shows an increasing trend with respect to changes in higher values of AR. The amount of enhancement in the average Nu value is 95% which is significant when the cases with lowest and highest aspect ratio are compared. The horizontal location of the rotating cone resulted in first deterioration of average heat transfer from x${}_{0}$ = 0.35 H to 0.5 H and then increment until x${}_{0}$ = 0.65 H, but the amount of variation is only 6% at the highest.

## 4. Conclusions

In the current work, impacts of a rotating cone with MHD effects are considered for the mixed convection of CNT-water nanofluid in a double lid-driven 3D trapezoidal cavity. Different behaviors of average heat transfer with respect to changes in the Richardson number is observed depending upon the angular rotational speed of the cone. The average Nusselt number increases for higher values of Richardson number when the cone is stationary and it shows a decreasing trend when the cone is rotating at speed of −300. Comparisons are also made between the 3D configuration with an inner rotating cone and 2D configuration with an inner rotating cylinder. Comparison results showed that the average Nusselt number is higher for 2D case. As the value of rotational speed increases, the discrepancy between the average Nu values between the 2D and 3D case and increases and highest value of 28.5% is obtained. Magnetic field effects reduced the effective convection but with the use of highly conductive CNT particles, the average Nu value enhances by about 84.3% at the highest magnetic field strength when the Hartmann number is taken as 50. The aspect ratio of the cone was found to be an effective parameter for heat transfer enhancement in 3D configuration and up to 95% average Nu value enhancements are observed when the values for lowest and highest aspect ratio are compared. However, the horizontal location of the rotating cone has a slight impact on the heat transfer.

## Figures and Tables

**Table 1:** Thermophysical properties of base fluid and nanoparticles [41].

Property	Water	SWCNT	MWCNT
$\rho \;(\mathrm{kg}/m^{3})$	997.1	2600	1600
$\;c_{p}\;\left(J/\mathrm{kg}\;K\right)$	4179	425	796
k(W/mK)	0.61	6600	3000

**Table 2:** Grid independence test results for three different values of Richardson numbers (Ha = 50, Da = 10${}^{-2}$, ω=−300, AR = 0.25, x${}_{0}$ = 0.5 H, ϕ=0.04).

Grid Name	Number of Elements	Nu (Ri = 0.05)	Nu (Ri = 1)	Nu (Ri = 50)
G1	1923	6.45	4.06	3.47
G2	4834	5.29	3.64	3.27
G3	7680	4.56	3.36	3.11
G4	19298	4.23	3.26	3.06
G5	39287	3.91	3.15	3.02
G6	69769	3.85	3.14	3.01
G7	69769	3.84	3.13	3.01

## Abbreviations/Nomenclature

		*x, y*	Cartesian coordinates
*AR*	aspect ratio	**Greek Characters**	
*F* ${}_{k}$	weight function	α	thermal diffusivity
Gr	Grashof number	β	expansion coefficient
*g*	gravitational acceleration	ϕ	solid volume fraction
*h*	local heat transfer coefficient	ν	kinematic viscosity
*Ha*	Hratamnn nunber	θ	non-dimensional temperature
*k*	thermal conductivity	ε	porosity
*n*	unit normal vector	κ	permeability
Nu${}_{s}$	local Nusselt number	ρ	density of the fluid
Nu${}_{m}$	average Nusselt number	σ	electrical conductivity
*p*	pressure	ω	rotational speed of the cone
Pr	Prandtl number	**Subscripts**	
*R*	normalized residual	*c*	cold
Ra	Rayleigh number	*CNT*	carbon nanotube
Re	Reynolds number	*h*	hot
Ri	Richardson number	*m*	average
*T*	temperature	*nf*	nanofluid
*u, v*	x-y velocity components	*p*	solid particle

## References

[1] Chamkha A.J., Abu-Nada E. (2012). Mixed convection flow in single- and double-lid driven square cavities filled with water—Al_2_O_3_ nanofluid: Effect of viscosity models. Eur. J. Mech. B Fluids.

[2] Islam A., Sharif M., Carlson E. (2012). Mixed convection in a lid driven square cavity with an isothermally heated square blockage inside. Int. J. Heat Mass Transf..

[3] Garmroodi M.R.D., Ahmadpour A., Talati F. (2019). MHD mixed convection of nanofluids in the presence of multiple rotating cylinders in different configurations: A two-phase numerical study. Int. J. Mech. Sci..

[4] Barnoon P., Toghraie D., Dehkordi R.B., Abed H. (2019). MHD mixed convection and entropy generation in a lid-driven cavity with rotating cylinders filled by a nanofluid using two phase mixture model. J. Magn. Magn. Mater..

[5] Lewis E. (1979). Steady flow between a rotating circular cylinder and fixed square cylinder. J. Fluid Mech..

[6] Khanafer K., Aithal S. (2013). Laminar mixed convection flow and heat transfer characteristics in a lid driven cavity with a circular cylinder. Int. J. Heat Mass Transf..

[7] Sheikholeslami M., Gorji-Bandpy M., Ganji D., Soleimani S., Seyyedi S. (2012). Natural convection of nanofluids in an enclosure between a circular and a sinusoidal cylinder in the presence of magnetic field. Int. Commun. Heat Mass Transf..

[8] Oztop H.F., Zhao Z., Yu B. (2009). Fluid flow due to combined convection in lid-driven enclosure having a circular body. Int. J. Heat Fluid Flow.

[9] Costa V.A.F., Raimundo A.M. (2010). Steady mixed convection in a differentially heated square enclosure with an active rotating circular cylinder. Int. J. Heat Mass Transf..

[10] Hussain S.H., Hussein A.K. (2011). Mixed convection heat transfer in a differentially heated square enclosure with a conductive rotating circular cylinder at different vertical locations. Int. Commun. Heat Mass Transf..

[11] Ghaddar N., Thiele F. (1994). Natural convection over a rotating cylindrical heat source in a rectangular enclosure. Numer. Heat Transf. A Appl..

[12] Fu W., Cheng C., Shieh W. (1994). Enhancement of natural convection heat transfer of an enclosure by a rotating circular cylinder. Int. J. Heat Mass Transf..

[13] Selimefendigil F., Oztop H.F. (2019). Conjugate mixed convection of nanofluid in a cubic enclosure separated with a conductive plate and having an inner rotating cylinder. Int. J. Heat Mass Transf..

[14] Kareem A.K., Gao S. (2018). A comparison study of mixed convection heat transfer of turbulent nanofluid flow in a three-dimensional lid-driven enclosure with a clockwise versus an anticlockwise rotating cylinder. Int. Commun. Heat Mass Transf..

[15] Selimefendigil F., Oztop H.F. (2018). Mixed convection of nanofluids in a three dimensional cavity with two adiabatic inner rotating cylinders. Int. J. Heat Mass Transf..

[16] Sarris I., Zikos G., Grecos A., Vlachos N. (2006). On the limits of validity of the low magnetic Reynolds number approximation in MHD natural-convection heat transfer. Numer. Heat Transf. Part B.

[17] Sajjadi H., Delouei A.A., Izadi M., Mohebbi R. (2019). Investigation of MHD natural convection in a porous media by double MRT lattice Boltzmann method utilizing MWCNT-Fe3O4/water hybrid nanofluid. Int. J. Heat Mass Transf..

[18] Geridonmez B.P., Oztop H.F. (2019). Natural convection in a cavity filled with porous medium under the effect of a partial magnetic field. Int. J. Mech. Sci..

[19] Wang Z.H., Zhou Z.K. (2019). External natural convection heat transfer of liquid metal under the influence of the magnetic field. Int. J. Heat Mass Transf..

[20] Rahman M., Oztop H.F., Saidur R., Mekhilef S., Al-Salem K. (2013). Finite element solution of MHD mixed convection in a channel with a fully or partially heated cavity. Comput. Fluids.

[21] Selimefendigil F., Oztop H.F. (2014). Numerical study of MHD mixed convection in a nanofluid filled lid driven square enclosure with a rotating cylinder. Int. J. Heat Mass Transf..

[22] Pekmen B., Sezgin M.T. (2014). MHD flow and heat transfer in a lid-driven porous enclosure. Comput. Fluids.

[23] Selimefendigil F., Oztop H.F. (2019). Fluid-solid interaction of elastic-step type corrugation effects on the mixed convection of nanofluid in a vented cavity with magnetic field. Int. J. Mech. Sci..

[24] Abbassi H., Nassrallah S.B. (2007). MHD flow and heat transfer in a backward-facing step. Int. Commun. Heat Mass Transf..

[25] Selimefendigil F., Oztop H.F. (2015). Influence of inclination angle of magnetic field on mixed convection of nanofluid flow over a backward facing step and entropy generation. Adv. Powder Technol..

[26] Ma Y., Mohebbi R., Rashidi M.M., Yang Z., Sheremet M.A. (2019). Numerical study of MHD nanofluid natural convection in a baffled U-shaped enclosure. Int. J. Heat Mass Transf..

[27] Selimefendigil F., Oztop H.F. (2016). Analysis of MHD mixed convection in a flexible walled and nanofluids filled lid-driven cavity with volumetric heat generation. Int. J. Mech. Sci..

[28] Mahmoudi A., Mejri I., Abbassi M.A., Omri A. (2015). Analysis of MHD natural convection in a nanofluid-filled open cavity with non uniform boundary condition in the presence of uniform heat generation/absorption. Powder Technol..

[29] Sheikholeslami M., Bandpy M.G., Ellahi R., Zeeshan A. (2014). Simulation of MHD CuO-water nanofluid flow and convective heat transfer considering Lorentz forces. J. Magn. Magn. Mater..

[30] Salman S., Talib A.R.A., Saadon S., Sultan M.T.H. (2020). Hybrid nanofluid flow and heat transfer over backward and forward steps: A review. Powder Technol..

[31] Javed S., Ali H.M., Babar H., Khan M.S., Janjua M.M., Bashir M.A. (2020). Internal convective heat transfer of nanofluids in different flow regimes: A comprehensive review. Phys. A Stat. Mech. Appl..

[32] Huminic G., Huminic A. (2020). Entropy generation of nanofluid and hybrid nanofluid flow in thermal systems: A review. J. Mol. Liq..

[33] Mahian O., Kianifar A., Heris S.Z., Wen D., Sahin A.Z., Wongwises S. (2017). Nanofluids effects on the evaporation rate in a solar still equipped with a heat exchanger. Nano Energy.

[34] Cho C.C. (2014). Heat transfer and entropy generation of natural convection in nanofluid-filled square cavity with partially-heated wavy surface. Int. J. Heat Mass Transf..

[35] Tiwari R., Das M.K. (2007). Heat Transfer Augmentation in a Two-Sided Lid-Driven Differentially Heated Square Cavity Utilizing Nanofluids. Int. J. Heat Mass Transf..

[36] Oztop H.F., Abu-Nada E. (2008). Numerical study of natural convection in partially heated rectangular enclosures filled with nanofluids. Int. J. Heat Fluid Flow.

[37] Koo J., Kleinstreuer C. (2005). Laminar nanofluid flow in microheat-sinks. Int. J. Heat Mass Transf..

[38] Hajialigol N., Fattahi A., Ahmadi M.H., Qomi M.E., Kakoli E. (2015). MHD mixed convection and entropy generation in a 3-D microchannel using Al2O3 water nanofluid. J. Taiwan Inst. Chem. Eng..

[39] Drummond J., Korpela S. (1987). Natural convection in a shallow cavity. J. Fluid Mech..

[40] Sheikholeslami M., Shehzad S.A., Li Z. (2018). Water based nanofluid free convection heat transfer in a three dimensional porous cavity with hot sphere obstacle in existence of Lorenz forces. Int. J. Heat Mass Transf..

[41] Al-Rashed A.A., Kalidasan K., Kolsi L., Aydi A., Malekshah E.H., Hussein A.K., Kanna P.R. (2018). Three-dimensional investigation of the effects of external magnetic field inclination on laminar natural convection heat transfer in CNT water nanofluid filled cavity. J. Mol. Liq..

[42] Ghasemi K., Siavashi M. (2020). Three-dimensional analysis of magnetohydrodynamic transverse mixed convection of nanofluid inside a lid-driven enclosure using MRT-LBM. Int. J. Mech. Sci..

[43] Gibanov N.S., Sheremet M.A., Oztop H.F., Al-Salem K. (2018). MHD natural convection and entropy generation in an open cavity having different horizontal porous blocks saturated with a ferrofluid. J. Magn. Magn. Mater..

[44] Miroshnichenko I.V., Sheremet M.A., Oztop H.F., Abu-Hamdeh N. (2018). Natural convection of alumina-water nanofluid in an open cavity having multiple porous layers. Int. J. Heat Mass Transf..

[45] Ghasemi K., Siavashi M. (2017). MHD nanofluid free convection and entropy generation in porous enclosures with different conductivity ratios. J. Magn. Magn. Mater..

[46] Raza J., Rohni A.M., Omar Z. (2016). MHD flow and heat transfer of Cu-water nanofluid in a semi porous channel with stretching walls. Int. J. Heat Mass Transf..

[47] Mehryan S.A.M., Sheremet M.A., Soltani M., Izadi M. (2019). Natural convection of magnetic hybrid nanofluid inside a double-porous medium using two-equation energy model. J. Mol. Liq..

[48] Kumaresan V., Velraj R. (2012). Experimental investigation of the thermo-physical properties of water-ethylene glycol mixture based CNT nanofluids. Thermochim. Acta.

[49] Selimefendigil F., Oztop H.F. (2019). Corrugated conductive partition effects on MHD free convection of CNT-water nanofluid in a cavity. Int. J. Heat Mass Transf..

[50] Abu-Nada E., Chamkha A.J. (2010). Mixed convection flow in a lid-driven inclined square enclosure filled with a nanofluid. Eur. J. Mech. B Fluids.

[51] Basak T., Roy S., Pop I. (2009). Heat flow analysis for natural convection within trapezoidal enclosures based on heatline concept. Int. J. Heat Mass Transf..

[52] Brinkman H. (1952). The viscosity of concentrated suspensions and solutions. J. Chem. Phys..

[53] Al-Sayegh R. (2018). Influence of external magnetic field inclination on three-dimensional buoyancy-driven convection in an open trapezoidal cavity filled with CNT-Water nanofluid. Int. J. Mech. Sci..

[54] Al-Rashed A.A., Kolsi L., Kalidasan K., Malekshah E.H., Kanna P.R. (2017). Second law analysis of natural convection in a CNT-water nanofluid filled inclined 3D cavity with incorporated Ahmed body. Int. J. Mech. Sci..

[55] Halelfadl S., Estelle P., Aladag B., Doner N., Mare T. (2013). Viscosity of carbon nanotubes water-based nanofluids: Influence of concentration and temperature. Int. J. Therm. Sci..

[56] Benos L.T., Karvelas E.G., Sarris I.E. (2019). Crucial effect of aggregations in CNT-water nanofluid magnetohydrodynamic natural convection. Therm. Sci. Eng. Prog..

[57] Razmara N., Namarvari H., Meneghini J.R. (2019). A new correlation for viscosity of model water-carbon nanotube nanofluids: Molecular dynamics simulation. J. Mol. Liq..

[58] Ouertatani N., Cheikh N.B., Beya B.B., Lili T., Campo A. (2009). Mixed convection in a double lid-driven cubic cavity. Int. J. Therm. Sci..

[59] Heyhat M.M., Kowsary F., Rashidi A.M., Momenpour M.H., Amrollahi A. (2013). Experimental investigation of laminar convective heat transfer and pressure drop of water-based Al_2_O_3_ nanofluids in fully developed flow regime. Exp. Therm. Fluid Sci..

[60] Oztop H.F., Dagtekin I. (2004). Mixed convection in two-sided lid-driven differentially heated square cavity. Int. J. Heat Mass Transf..

[61] Kaneda M., Tagawa T., Ozoe H. (2006). Natural convection of liquid metal under a uniform magnetic field with an electric current supplied from outside. Exp. Therm. Fluid Sci..

[62] Sawada T., Kikura H., Saito A., Tanahashi T. (1993). Natural convection of a magnetic fluid in concentric horizontal annuli under nonuniform magnetic fields. Exp. Therm. Fluid Sci..

[63] Li Q., Xuan Y. (2009). Experimental investigation on heat transfer characteristics of magnetic fluid flow around a fine wire under the influence of an external magnetic field. Exp. Therm. Fluid Sci..

[64] Sarafraz M.M., Hormozi F., Nikkhah V. (2016). Thermal performance of a counter-current double pipe heat exchanger working with COOH-CNT/water nanofluids. Exp. Therm. Fluid Sci..

[65] Rathnakumar P., Iqbal S.M., Michael J.J., Suresh S. (2018). Study on performance enhancement factors in turbulent flow of CNT/water nanofluid through a tube fitted with helical screw louvered rod inserts. Chem. Eng. Process. Process Intensif..

